# Cerebral oxygenation and perfusion kinetics monitoring of military aircrew at high G using novel fNIRS wearable system

**DOI:** 10.3389/fnrgo.2024.1357905

**Published:** 2024-02-23

**Authors:** Thibault Roumengous, R. Casey Boutwell, Jason Strohmaier, Jared Allen, Brett Goldbach, Nicholas Marotta, Tanner Songkakul, Shelby Critcher, Bria G. Morse, Jeremy M. A. Beer, Paul M. Sherman

**Affiliations:** ^1^NIRSense Inc., Richmond, VA, United States; ^2^Aerospace Environment Protection Lab, KBR Science and Space Government Solutions Group, San Antonio, TX, United States; ^3^USAF 59th Medical Wing Science and Technology, San Antonio, TX, United States

**Keywords:** deoxygenation kinetics, tissue oximetry, hypergravity, high-performance aircraft, decompensation, physiological episode detection, functional near-infrared spectroscopy, continuous monitoring

## Abstract

**Introduction:**

Real-time physiological episode (PE) detection and management in aircrew operating high-performance aircraft (HPA) is crucial for the US Military. This paper addresses the unique challenges posed by high acceleration (G-force) in HPA aircrew and explores the potential of a novel wearable functional near-infrared spectroscopy (fNIRS) system, named NIRSense Aerie, to continuously monitor cerebral oxygenation during high G-force exposure.

**Methods:**

The NIRSense Aerie system is a flight-optimized, wearable fNIRS device designed to monitor tissue oxygenation 13–20 mm below the skin's surface. The system includes an optical frontend adhered to the forehead, an electronics module behind the earcup of aircrew helmets, and a custom adhesive for secure attachment. The fNIRS optical layout incorporates near-distance, middle-distance, and far-distance infrared emitters, a photodetector, and an accelerometer for motion measurements. Data processing involves the modified Beer-Lambert law for computing relative chromophore concentration changes. A human evaluation of the NIRSense Aerie was conducted on six subjects exposed to G-forces up to +9 Gz in an Aerospace Environmental Protection Laboratory centrifuge. fNIRS data, pulse oximetry, and electrocardiography (HR) were collected to analyze cerebral and superficial tissue oxygenation kinetics during G-loading and recovery.

**Results:**

The NIRSense Aerie successfully captured cerebral deoxygenation responses during high G-force exposure, demonstrating its potential for continuous monitoring in challenging operational environments. Pulse oximetry was compromised during G-loading, emphasizing the system's advantage in uninterrupted cerebrovascular monitoring. Significant changes in oxygenation metrics were observed across G-loading levels, with distinct responses in Deoxy-Hb and Oxy-Hb concentrations. HR increased during G-loading, reflecting physiological stress and the anti-G straining maneuver.

**Discussion:**

The NIRSense Aerie shows promise for real-time monitoring of aircrew physiological responses during high G-force exposure. Despite challenges, the system provides valuable insights into cerebral oxygenation kinetics. Future developments aim for miniaturization and optimization for enhanced aircrew comfort and wearability. This technology has potential for improving anti-G straining maneuver learning and retention through real-time cerebral oxygenation feedback during centrifuge training.

## 1 Introduction

A physiological episode (PE) refers to a pilot experiencing loss in performance due to flight-related environmental stressors. Real-time PE detection and management in aircrew operating high-performance aircraft (HPA) is a priority focus area for all Air Force worldwide that have HPA, the US Military in particular. Unique occupational hazards of HPA aircrew include sustained high acceleration, or G-force, in combination with low cockpit pressure and varying oxygen delivery schedules intended to support aircrew physiological demand during rapid changes in flight operations (Bouak et al., 2018; Shaw et al., 2021). Because of the high cognitive and physical capacity required by HPA during combat jet maneuvers, it is important to address the effects of repeated and acute G-exposure on both immediate and long-term operational performance (Hormeño-Holgado and Clemente-Suárez, 2019). Thus, physiological monitoring technology has the potential to help better detect, diagnose, and counteract PEs, but integration into military aviation platforms presents challenges with respect to current sensor technology availability and integration limitations (Shaw and Harrell, 2023).

Electroencephalography (EEG) systems have been used to measure brain activity and cognitive workload, helping understand the mental demands placed on pilots during different phases of flight (Villafaina et al., 2021; Masi et al., 2023). However, current EEG systems have poor spatial resolution, are highly sensitive to vibration artifacts, and cannot inform about the state of the cerebrovascular system which is primarily affected when rapid changes in gravitational force occur during HPA flight.

Near-infrared spectroscopy (NIRS) is another method that has been suggested to be capable of measuring brain activity-related physiological changes via noninvasive monitoring of oxygenation and deoxygenation of tissues underneath the sensor (Madsen and Secher, 1999). While reduced brain oxygenation due to decreased cerebral perfusion is expected to occur under hypergravity (Pollock et al., 2021), prolonged G-exposure effects on prefrontal cortex (PFC) oxygenation have been inconsistent in past reports (Kobayashi and Miyamoto, 2000; Kobayashi et al., 2002; Smith et al., 2013; Konishi et al., 2019). Due to historical sensing limitations, there is limited knowledge about the deoxygenation kinetics responses of the brain during repeated, acute G-exposure, especially high G-exposure (> 5G).

A wearable flight-optimized system for continuous cerebral oximetry, the NIRSense Aerie, was designed for aircrew physiological monitoring and evaluated for the potential to detect Pes in HPA. The NIRSense wearable functional NIRS (fNIRS) system, in addition to measuring forehead pulse oximetry signals using photoplethysmography (PPG), uses NIRS to noninvasively measure concentration changes in both oxygenated and deoxygenated hemoglobin (Oxy-Hb and Deoxy-Hb) in a region of tissue ~13–20mm below the skin's surface, deep enough to obtain reflected light that interacted with cerebral tissue. Simultaneously recording NIRS and PPG allows for comprehensive monitoring of tissue oxygenation and perfusion of aircrew in challenging operational environments that affect both the ambient sensor environment and the underlying aircrew physiology.

The NIRSense Aerie system was implemented in a human centrifuge acceleration protocol conducted for the US Air Force. This human evaluation (Aerospace Environmental Protection Laboratory (AEPL), San Antonio, TX) was conducted to identify critical physiological and psychomotor responses that occur throughout a proven acceleration training curriculum to optimize the gain realized in those components. This report aims to describe the novel wearable fNIRS system designed for HPA aircrew population and present preliminary data collected in HPA-relevant recording environments.

## 2 Materials and methods

### 2.1 Wearable fNIRS system description

#### 2.1.1 System overview

**Figure 1:**
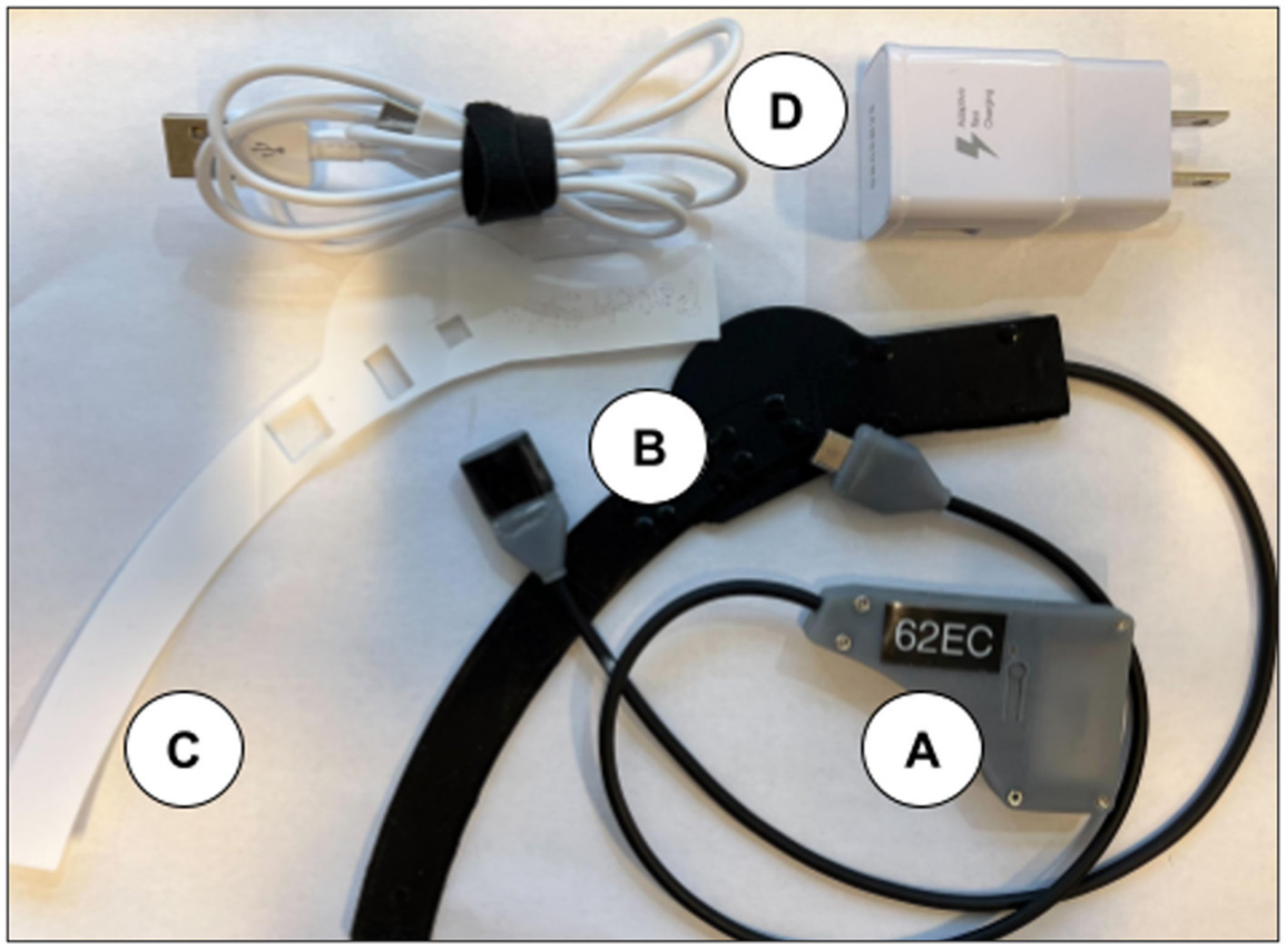
NIRSense Aerie electronics module **(A)**, forehead optical patch **(B)**, custom adhesive **(C)**, and USB-C charging and cables **(D)**. The USB-C Charger is a commercial off-the-shelf (COTS) battery charger for the electronics module.

The NIRSense Aerie wearable fNIRS system is designed to monitor tissue oxygen 13–20 mm below the skin's surface and is encapsulated in medical silicone. The fNIRS optical frontend (Figure 1B) adheres to the skin using a custom two-layer adhesive (Figure 1C) using biocompatible, hypoallergenic 3M materials. The Aerie optical frontend is the forehead-worn sensor component of the NIRSense Aerie system and connects to the electronics module via a USB-C connector. The wired electronics module (Figure 1A), is designed to fit and be repositionable behind the ear cup of aircrew helmets, stores data securely onboard, and communicates over Bluetooth to a companion mobile application. The Aerie electronics module consists of the core NIRSense system electronics and battery. It is connected to the forehead-worn optical frontend via a cable connection. The optical signals captured on the optical frontend are digitized and processed by the electronics module and stored in onboard memory for post-processing as well as transmitted over Bluetooth Low Energy to the companion application. The forehead frontend has an integrated haptics motor for real-time aircrew alerts. Bluetooth can be disabled for autonomous operation. The battery can be charged using a commercial-off-the-shelf (COTS) USB-C charger and cable (Figure 1D) and can last for up to 13 h of continuous use. The system's technical specifications are summarized in Table 1.

**Table 1:** NIRSense Aerie fNIRS system technical specifications.

Dimensions	193.0 × 61.1 × 5.5 (patch, mm)
	64.9 × 41 × 8.5 (electronics module, mm)
Weight	28g (patch)
	28g (electronics module)
Optics	Red/near-infrared LEDs
Sensors	Silicone Photomultiplier (SiPM)
	3-axis Accelerometer
Wavelengths	740 nm
	850 nm
	940 nm
Penetration depth	13–20 mm (deep tissue optics)
	11–17.5 mm (middle distance tissue optics)
	5–7.5 mm (shallow tissue optics)
Body design	Patch: RTV 1556 silicone encapsulation
	electronics module: Tough Resin 1500 encapsulation
Adhesive design	3M 2480 (skin-side interface)
	3M 96042 (device-side interface)
Battery life	Up to 13 h
Battery capacity	250 mAh
Operating voltage (system)	3.7 V
Operating voltage (SiPM)	28 V
Nominal operating current	19.2 mA
Sampling frequency	25–50Hz
Communication method	Bluetooth Low Energy (BLE) v5.2
Communication modes	Real-Time Streaming
	Autonomous Untethered (BLE Off)
Software	NIRSense Windows application
Charging method	USB-C (5V)
Charging time	~90 min

#### 2.1.2 System mechanical design

**Figure 2:**
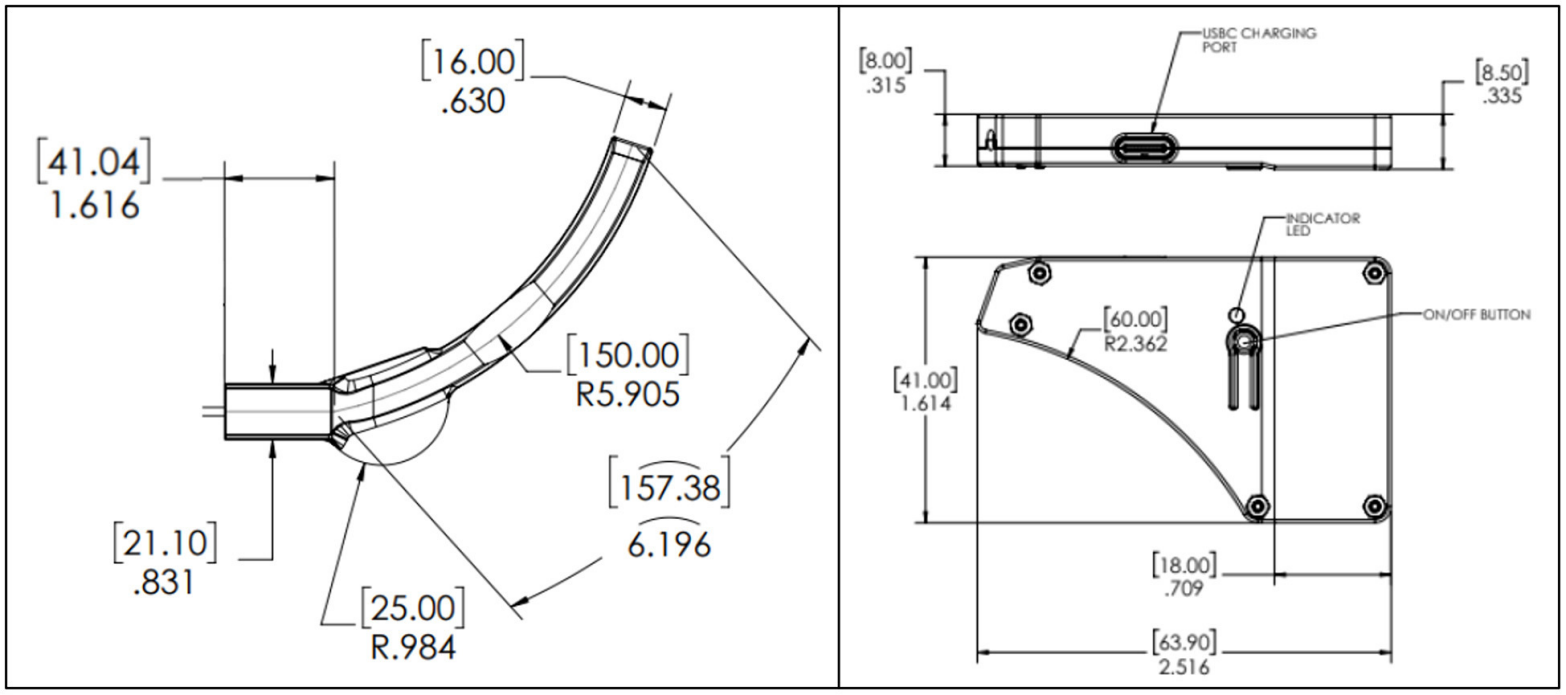
NIRSense Aerie optical frontend **(left)** and electronics module **(right)** detailed mechanical design optimized for HPA aircrew population. Measurements are reported in mm (brackets) and inches, according to the engineering drawing standards (ISO 8015).

**Figure 3:**
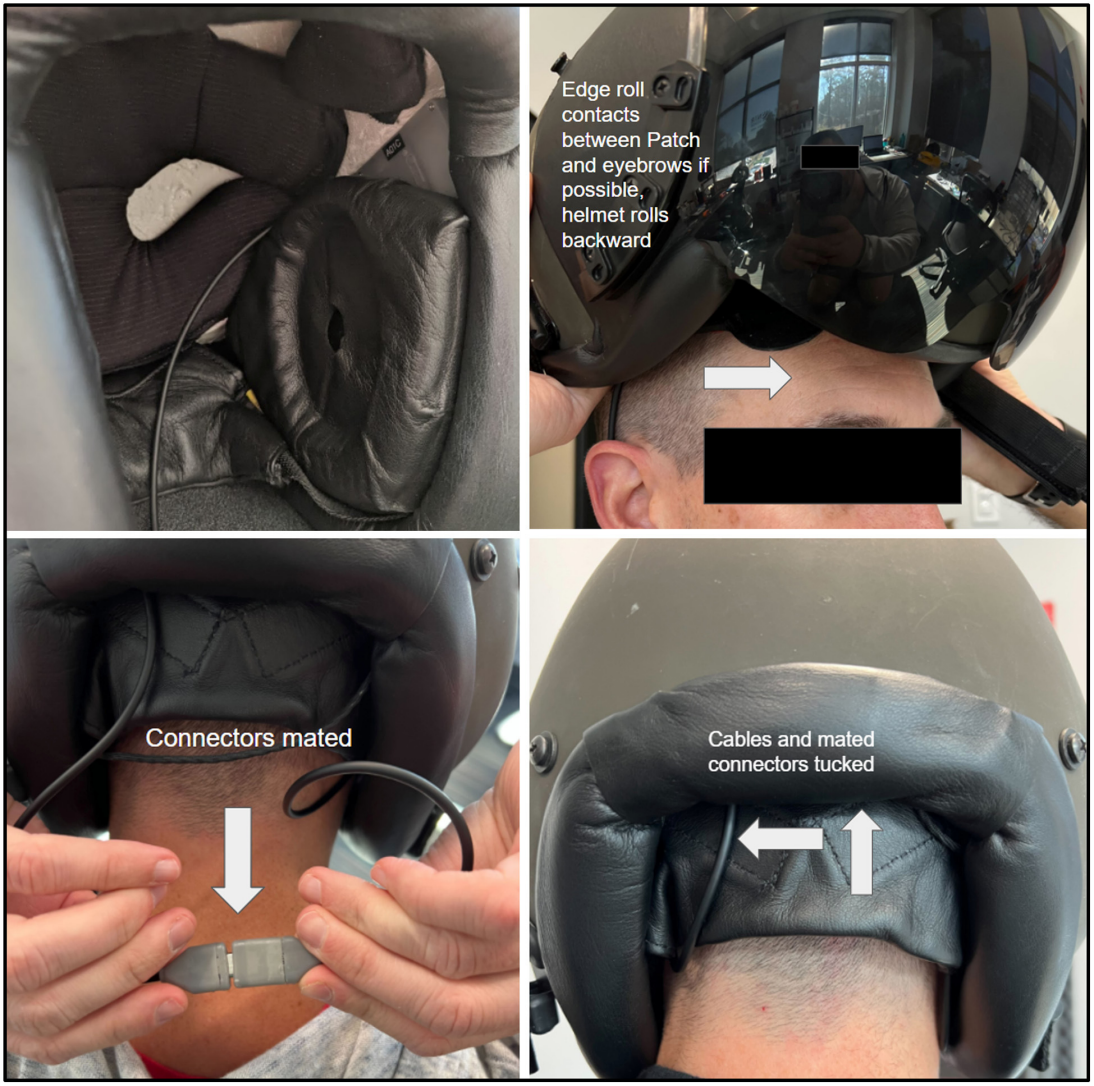
NIRSense Aerie system aircrew setup when operated under a helmet. The optical frontend is designed to fit under the edge roll while the electronics module fits behind the earcup.

The Aerie fNIRS system enclosure was specifically designed to meet the human factors requirements of military HPA users. The optical frontend consists of a fully flexible and ultra-thin patch encapsulated in silicone that can fit under the edge roll of an aircrew's helmet. The electronics module's thin, compact design allows it to fit behind the earcup of the aircrew's helmet. Figure 2 shows the engineering drawing of both the optical frontend and the electronics module. Figure 3 shows the system setup for an aircrew user with positioning of the electronics module and the optical frontend when worn under an HPA aircrew's helmet. The following sections provide detailed descriptions of the system and its components.

#### 2.1.3 Aerie optical frontend

The NIRSense Aerie system includes an ultra-thin optical frontend which is attached to the forehead via an adhesive patch and enables measurement of both superficial and cerebral tissue oxygenation metrics. The optoelectronic sensor subsystem generates optical data that is transmitted to, and processed by, the electronics module. It allows for configurable haptic feedback to alert the user based on the calculated oxygenation data and user-defined threshold alert conditions. The frontend flexible enclosure is cast in black dyed room temperature vulcanized silicone rubber and its dimensions are ~5.5 mm (maximal thickness) × 193.0 mm (length) × 61.1 mm (width). The center of the optical front end is < 2 mm thick. The optical frontend of the Aerie fNIRS system was designed to meet the human factors requirements of the military HPA population with respect to usability, comfort, and compatibility with existing equipment.

##### 2.1.3.1 Flexible-printed circuit board

**Figure 4:**
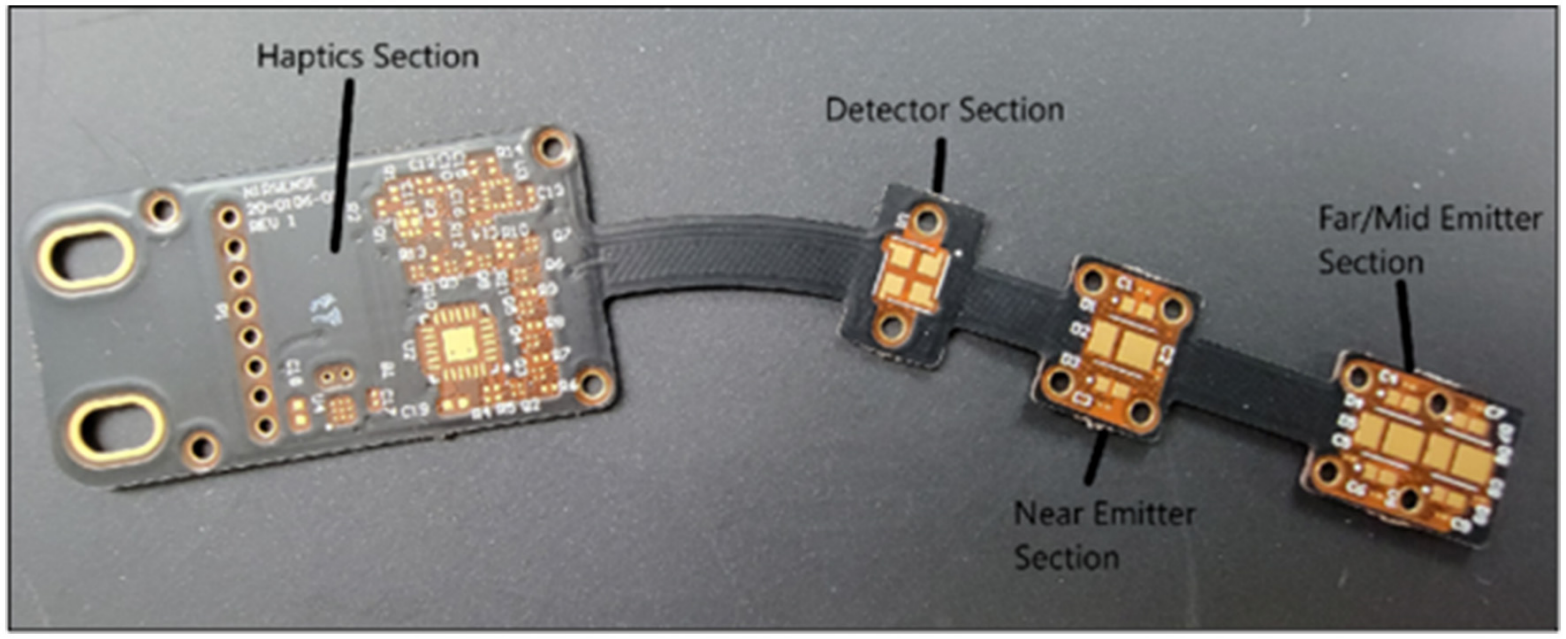
Optical Frontend Flexible PCB Board, highlighting the haptics, detector and emitters sections.

The PCB (Figure 4) within the optical frontend is required to house the emitter and detector optics, the accelerometer, and the haptic motor. The PCB has three flex regions to allow flexibility to conform around the forehead. The black color of the solder mask along with black EMI film laminated on top of the cover is designed to prevent undesired optical reflections, while also providing electrical shielding of PCB signal layers. The flex circuit layers consist of polyimide core, adhesive, and copper layers.

##### 2.1.3.2 Silicone lens

The silicone lens is required to cover the skin-contacting side of the optical frontend to ensure user comfort and provide a translucent light guide between the optics and the skin. It is made of RTV silicone and conforms to the shape of the optical frontend to improve long-term wearability.

##### 2.1.3.3 fNIRS optical layout

The fNIRS optical layout is composed of a photodetector, near-distance infrared emitters, middle-distance infrared emitters, and far-distance infrared emitters. The distances between photodetector and each of the three emitter banks are 15 mm, 35 mm, and 40 mm, respectively. The near, middle, and far distance emitters emit light at three wavelengths: 740 nm, 850 nm, and 940 nm. A highly sensitive, low-noise, Silicon Photomultiplier (SiPM) is used as a photodetector to measure the light scattered back from the tissue underneath the sensor across nine measurement channels (three distances, three wavelengths), and one ambient light measurement channel.

##### 2.1.3.4 Accelerometer

The accelerometer of the Aerie system is designed into the flexible printed circuit board for acceleration (i.e. G-force) and motion (i.e. human motion artifacts, vibrations) measurements. The optical frontend patch PCB includes a tri-axis accelerometer (Kionix Inc) with a range of ±16G and an embedded low pass filter. The accelerometer is 2 mm x 2 mm x 0.9 mm.

#### 2.1.4 Electronics module

The electronics module was designed to be integrated into the helmet behind the left earcup. It can be secured to the helmet via a hook and loop strip on the outside of the electronics module (Figure 3) and compression fit between the earcup and interior white foam region of the helmet. The power cable is routed to the back of the helmet near the nape strap and connected to the Aerie Patch frontend cable. The electronics module enclosure is 3D printed. The electronics module's dimensions are 8.5 mm (W) x 63.9 mm (L) x 41 mm (H). The electronics module weighs ~26.0 g.

##### 2.1.4.1 Rigid-printed circuit board

The rigid PCB (within the electronics module in Figure 3) consists of interleaved layers of copper with glass-reinforced epoxy laminate and a black solder mask. The rigid PCB assembly contains all of the non-optical core electronics, including the main microcontroller, flash memory, and battery/power management hardware.

##### 2.1.4.2 Flash memory

The data are transferred from the flash memory to a NIRSense Windows software application post-session via Bluetooth Low Energy (BLE). Data are stored within a 2 GB flash memory, which was chosen to operate at a wide temperature range.

##### 2.1.4.3 Battery pack

The Aerie requires a single-cell rechargeable lithium polymer battery rated at 250 mAh as the power source for the device. The battery provides about 13 h of runtime for the patch in the current device configuration. The battery is UL1642 listed and IEC62133 certified. The battery can be charged using a COTS USB-C battery charger. When charging is complete, the charger discontinues applying charge to the battery. The time to recharge the battery pack using the provided single-channel charger is < 90 min from near depletion.

#### 2.1.5 Adhesive

The custom-cut 3M adhesive laminate is a combination of 3M's 2480 hypoallergenic skin contact adhesive and 96042 silicone adhesive. The skin-side adhesive is silicone-based and has a polypropylene liner; the Patch side is a silicone-based adhesive covered with a polyethylene terephthalate (PET) liner. The total thickness of the tape is 0.55 mm with the two adhesives contributing to 0.46 mm of overall thickness. This 3M combination adhesive is a breathable solution for device attachment on a user experiencing high sweat conditions; the skin adhesive uses a nonwoven spunlace carrier covered with a transparent liner. The device-side adhesive is a strong industrial and biocompatible silicone 3M tape.

#### 2.1.6 System signal optimization and processing

The NIRSense Aerie uses a 16-bit Analog-to-Digital Converter (ADC) to sample each of the nine measurement channels and one ambient light channel, as well as accelerometer data (3 channels) at 50 Hz. The Aerie captures optical densities (OD) from three backscattered wavelengths, each represented at three different measurement depths using LED banks positioned 15 mm, 35 mm, and 40 mm from the photodetector. At the start of sampling, the Aerie autonomously adjusts commanded currents to each emitter to optimize signal-to-noise ratio based on the recording environment and user's skin tone. During sampling, the gain of the SiPM is adjusted dynamically within the acquisition sequence to optimize the signal-to-noise ratio between the short channel (15 mm LEDs) and the long channels (35 mm, 40 mm LEDs). Finally, onboard processing is applied to compute pulse rate and oxygen saturation (SpO_2_) from the near (15 mm) channels (740 nm and 940 nm) as well as relative chromophore concentration change in the middle (35 mm) and far (40 mm) channels (740 and 850 nm) using the modified Beer-Lambert law (mBBL) (Kocsis et al., 2006).

### 2.2 Human centrifuge study

#### 2.2.1 Overview

**Figure 5:**
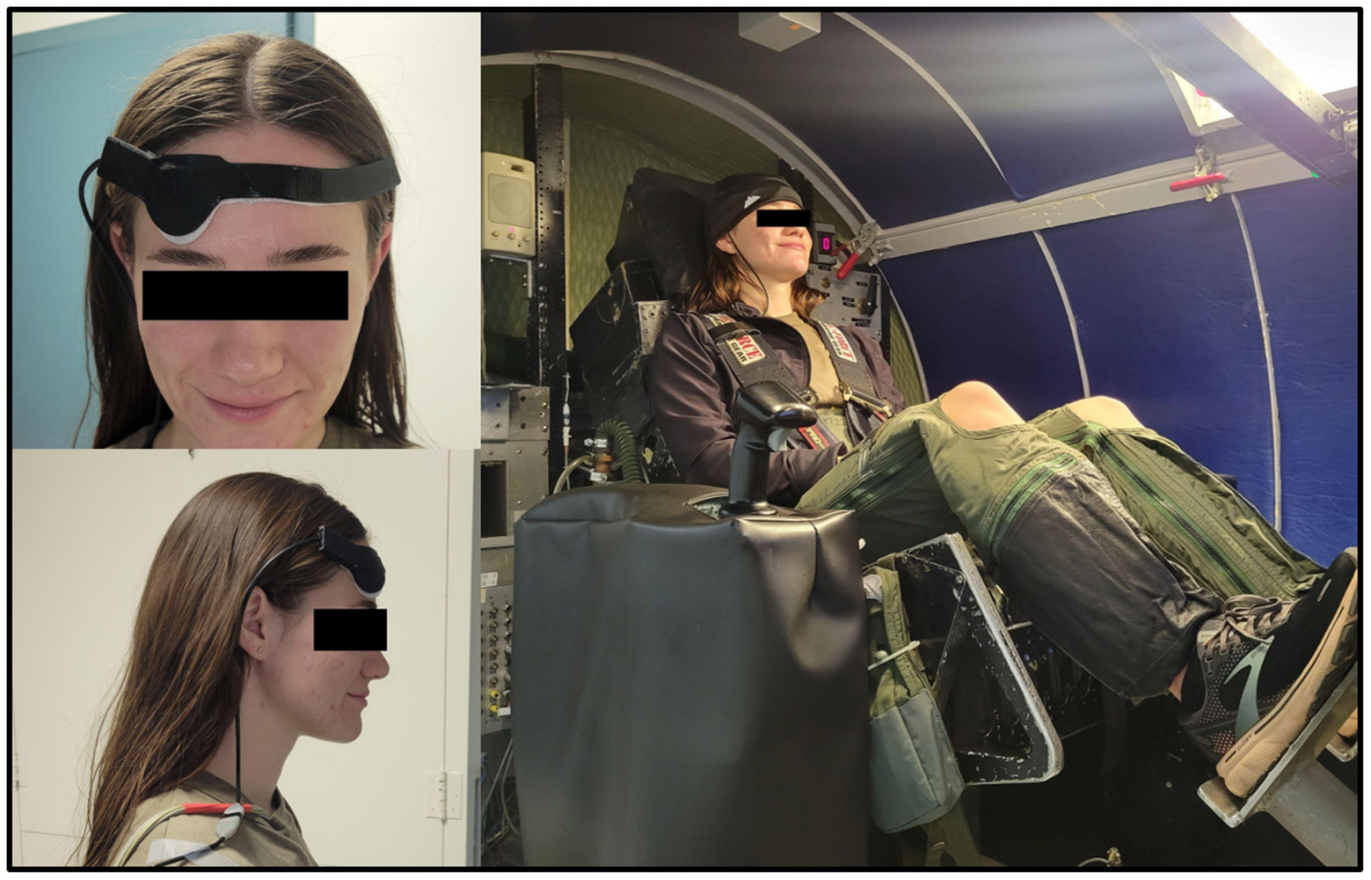
Human Centrifuge Study subject setup at the KBR facility in San Antonio, TX, USA.

Six subjects received training in the AEPL centrifuge up to a maximum of +9 Gz with the seat tilted back 30°. Subjects were briefed and provided their informed consent to Institutional Review Board (IRB)-approved protocols through Air Force Research Lab (AFRL) for human-rated centrifuge evaluation at KBR's Aerospace Environmental Protection Laboratory (AEPL) located in San Antonio, TX (AFRL IRB #FWR20220101H). Subjects were trained to successfully complete Rapid Onset Runs (ROR) (6.0 G/s onset rate) using a combination of G-suit and anti-G straining maneuver (AGSM). Both experienced centrifuge volunteers and new candidates were included in this study. Chest heart rate (HR) was collected using a Polar H10 monitor (Polar Electro Oy, Finland). NIRSense Aerie sensor was placed on the subject's right forehead (Figure 5).

#### 2.2.2 fNIRS data processing

Raw data was saved on the software application (NIRSense Windows App, v1.0.0.41) and the mBLL was applied as post-processing on the raw data collected during each session to compute fNIRS outputs. The mBLL states that optical density (OD) is the light attenuated after being backscattered and is proportional to changes in the concentration of chromophores in tissue (i.e., Oxy-Hb and Deoxy-Hb). Using a linear equation for two wavelengths, it is possible to solve for the relative concentration change of Deoxy-Hb and Oxy-Hb (Kocsis et al., 2006; Ayaz et al., 2011). To find the change in OD (ΔOD) of each wavelength (850 nm and 740 nm), the log difference between any given OD sample and a reference OD value obtained during the baseline period was computed for each wavelength. The differential pathlength factor (DPF) was estimated following an empirical formula that takes into account subject age and wavelength (Scholkmann and Wolf, 2013). Finally, relative change in total oxygenation was obtained by subtracting Deoxy-Hb from Oxy-Hb. Digital filtering was applied to eliminate the undesirable noise and fast variations that are not of interest to measuring the relative trend change of oxygenation. A sixth-order Butterworth low-pass filter was applied to the signals with a cut-off frequency of 0.5 Hz. Finally, a 10-second time window rolling average is applied using the convolution method.

#### 2.2.3 Data analysis

After data processing was applied as described in 2.2.2, datasets were inspected for signal quality and potential recording artifacts and noise such as ambient light noise, optical channel saturation, and power noise contamination. No major artifacts or noise were observed. Next, all datasets were trimmed to select only 15 second exposures to 5G, 7G, and 9G. Using onboard accelerometer data collected during the sessions, scripts were written to determine time of G-loading onset, peak-loading, and post-G recovery periods and subsequently extract mean values of each relative chromophore concentration (Oxy-Hb, Deoxy-Hb) at three distances. Mean oxygenation values immediately before G-loading (10 s window), mean values during peak G-exposure (5s window), and mean values during the recovery phase (20 s post G-loading) were extracted. Slopes of the total oxygenation data were calculated for the pre-G-loading to peak-G-loading. HR data was also extracted using the same logic.

Relative Chromophore concentration data were analyzed in three two-way ANOVA models (one per G-loading level) to test the effect of G-loading on Deoxy-Hb and Oxy-Hb across 3 detector channels (40, 35, 15 mm). Another two-way ANOVA was used to compare HR data between baseline, pre-G-loading, peak-G-loading, and post-G-loading conditions. ANOVA assumptions were tested using Shapiro-Wilk normality tests and visually inspecting residuals. Tuckey's HSD multiple comparison tests were used for *post hoc* analyses. Finally, oxygenation slopes data across G-levels were analyzed in a mixed-effect model with G condition (loading or recovery) and G level (5G, 7G, 9G) as fixed variables while subject # was set as random variable. Multiple comparisons were performed using Bonferroni's test. All data are presented as mean ± standard error of the mean unless stated otherwise. Statistical significance was set at the p < 0.05 level. Data analyses were performed using custom-written scripts in Python. Statistical analyses were performed using GraphPad Prism version 10.0.0 for Windows (GraphPad Software, Boston, Massachusetts USA), and R Statistical Software (v4.1.2; R Core Team, 2021).

## 3 Results and discussion

### 3.1 Overview

The NIRSense Aerie sensor successfully collected data on six subjects across up to four centrifuge sessions per subject. A total of 19 exposures at 5G, 15 exposures at 7G, and 10 exposures at 9G were conducted across all six subjects. No sensor decoupling occurred throughout the experiments. Subjective user feedback on system fit and feel was generally positive (agree/strongly agree to comfort). Inter-subject variability in physiological outcomes presented is relatively high but this is expected to be due to inclusion of both novices and highly experienced aircrew subjects into the same dataset. Group differences between AGSM experienced and novices were intentionally excluded from the scope of this report and will be presented elsewhere.

### 3.2 Cerebral and pulse oximetry outcomes

**Figure 6:**
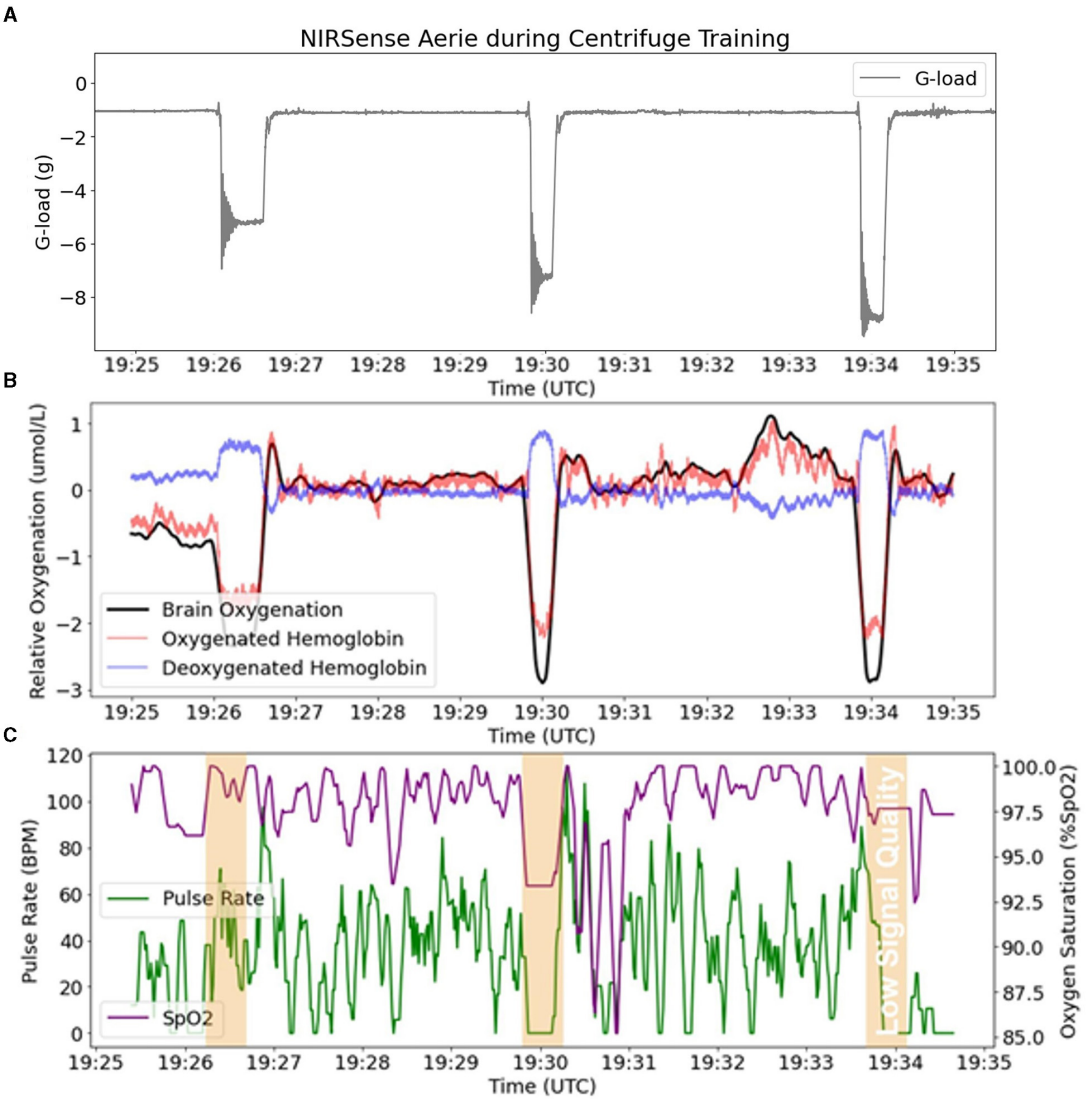
fNIRS outputs (Aerie sensor) compared to pulse oximetry outputs (Aerie sensor) during three successive G-loading events of increasing intensity. **(A)** Acceleration recorded by the Aerie sensor during three G exposures. **(B)** fNIRS outputs (Oxy-Hb, Deoxy-Hb, and Oxygenation) recording continuously. **(C)** The pulsatile component in the shallow signals was lost during G-loading, and thus limited the calculation of SpO_2_ and PR.

An example of representative fNIRS data along with Pulse Oximetry (PR and SpO_2_) data collected during 5G, 7G, and 9G back-to-back exposures is presented in Figure 6. Pulse Oximetry signal quality dramatically decreased during G-loading as illustrated in the bottom panel of Figure 6. This observation is consistent with a major decrease in superficial forehead tissue perfusion as a result of positive acceleration exposure. As the blood in the extracerebral vasculature is moved under the effect of positive acceleration, the pulsatile component in the PPG signals disappears which results in an inability to record accurate PR and SpO_2_ readings during G-loading. The pulsatile component recovers 5–10 s after the abatement of G-loading. Additionally, cerebral oxygenation metrics such as total Hb, Oxygenation, and Oxy-Hb were immediately decreased during G-loading while Deoxy-Hb concentrations were increased. This acute response is comparable to observations from previous research; a recent case study on two experienced participants in a human centrifuge showed trend oxygenation reduction (Lange et al., 2020). Another human centrifuge study found similar outcomes (although a different type of NIRS sensor was used, and G-exposure was milder and longer) (Smith et al., 2013) suggesting that the change in cerebral oxygenation metrics is likely due to acute reduction in cerebral perfusion during G-exposure. Smith et al. also showed that this effect was not related to brain activity (as measured by EEG). A recovery of the oxygenation parameters can also be observed immediately following the abatement of G-loading where overcompensation (hyperemia phase) occurs for 10–20 s before settling back to their pre-exposure baseline values. In the presence of hypergravity, these signal changes cannot be attributed to the neurovascular coupling effect typically measured by fNIRS to monitor brain activity (Tachtsidis and Scholkmann, 2016).

**Figure 7:**
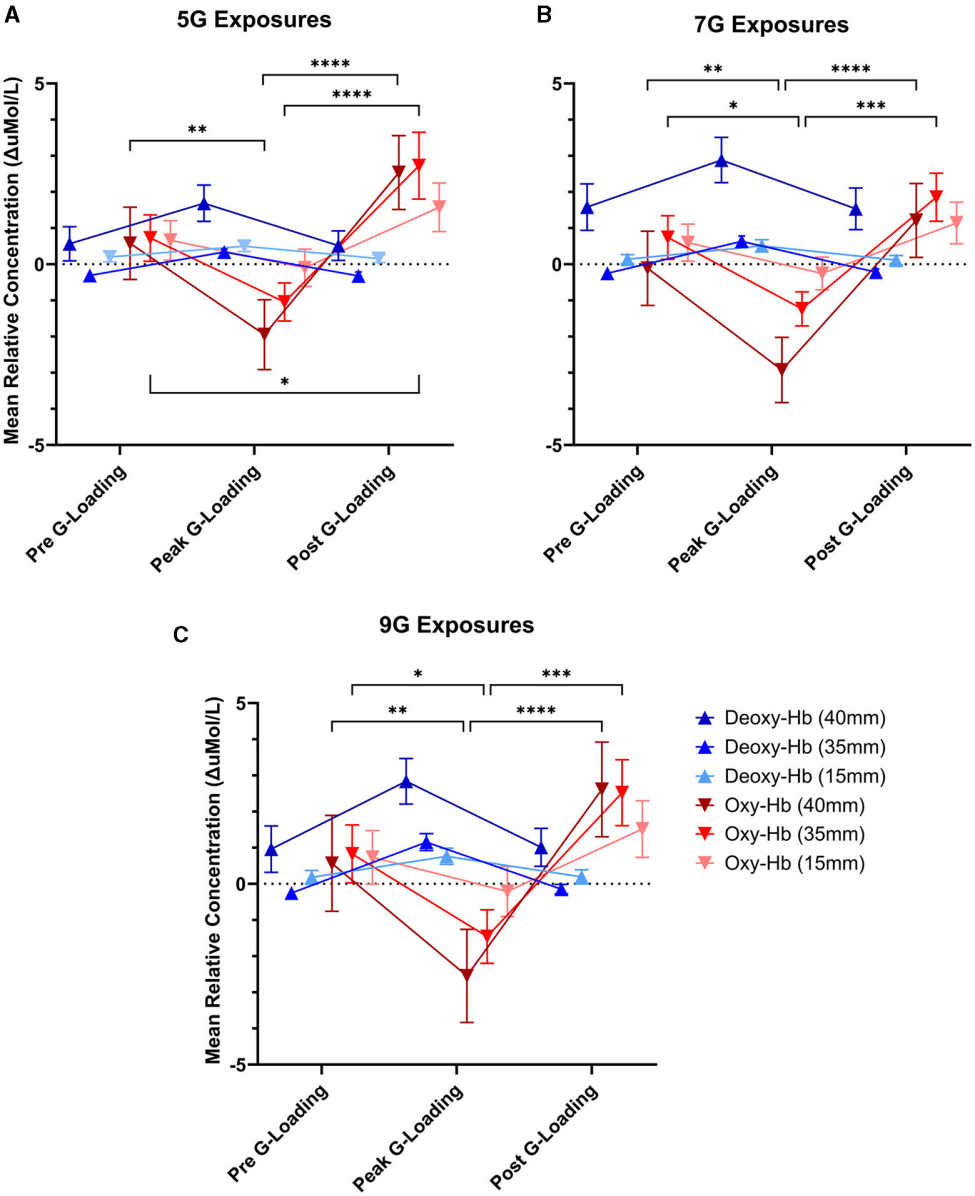
Mean relative concentration changes of deoxygenated and oxygenated hemoglobin at three time points: immediately pre-G-loading, at the peak of G-loading, and during recovery post-G-loading across three G-exposure intensities [**(A)** 5G, **(B)** 7G, and **(C)** 9G]. Error bars represent the standard error of the mean. Data are presented for all three fNIRS channels, 40mm, 35, and 15mm inter-probe distances, respectively. Asterisks indicate a significantly difference at the group level ([*] = *p* < 0.05, [**] = *p* < 0.01, [***] = *p* < 0.001, [****] = *p* < 0.0001).

There was a significant main effect of G-loading at 5G [F_(2, 324)_ = 7.342, *p* = 0.0008], 7G [F_(2, 252)_ = 4.564, *p* = 0.0113], and 9G [F_(2, 162)_ = 3.939, *p* = 0.0214] as well as a significant interaction effect between G-loading and Deoxy-Hb, Oxy-Hb across the three detector channels at 5G [F_(10, 324)_ = 4.253, *p* < 0.0001], 7G [F_(10, 252)_ = 4.090, *p* < 0.0001], and 9G [F_(10, 162)_ = 3.971, *p* < 0.0001]. Opposite trend responses to acute G-loading in Deoxy-Hb and Oxy-Hb concentration were observed across all measurement channels and G-exposure levels (Figure 7). During the G-loading phase, Deoxy-Hb was increased while Oxy-Hb was decreased. The reverse occurs during the recovery phase where Deoxy-Hb was decreased momentarily while Oxy-Hb was increased compared to their initial values. However, at the group level, *post hoc* tests revealed only significant changes in Oxy-Hb across all G-levels. This may be due to the generally smaller size of the Deoxy-Hb response to G-loading compared to Oxy-Hb along with the variability in relative measures between subjects. The recovery phase response was similar to those observed during vascular occlusion tests where tissue oxygenation recovers past its baseline value temporarily following induced ischemia (Futier et al., 2011). This hyperemia phase is likely related to vascular adaptation during G-loading leading to hypoxia-induced vasodilation that persists momentarily during reperfusion (Gourley and Heistad, 1984; Pollock et al., 2021). At the group level, significant differences between the pre-G-loading and the post-G-loading phase were only found at 5G and in the 35 mm detector channel (Figure 7A). Further, despite similar trend responses, the multi-depth measurements revealed no significant differences in the 15 mm detector channels (extracerebral compartment only). This is expected, as optical signals from the deeper channels correspond to light having interacted with more tissue, including within the cerebral compartment.

**Figure 8:**
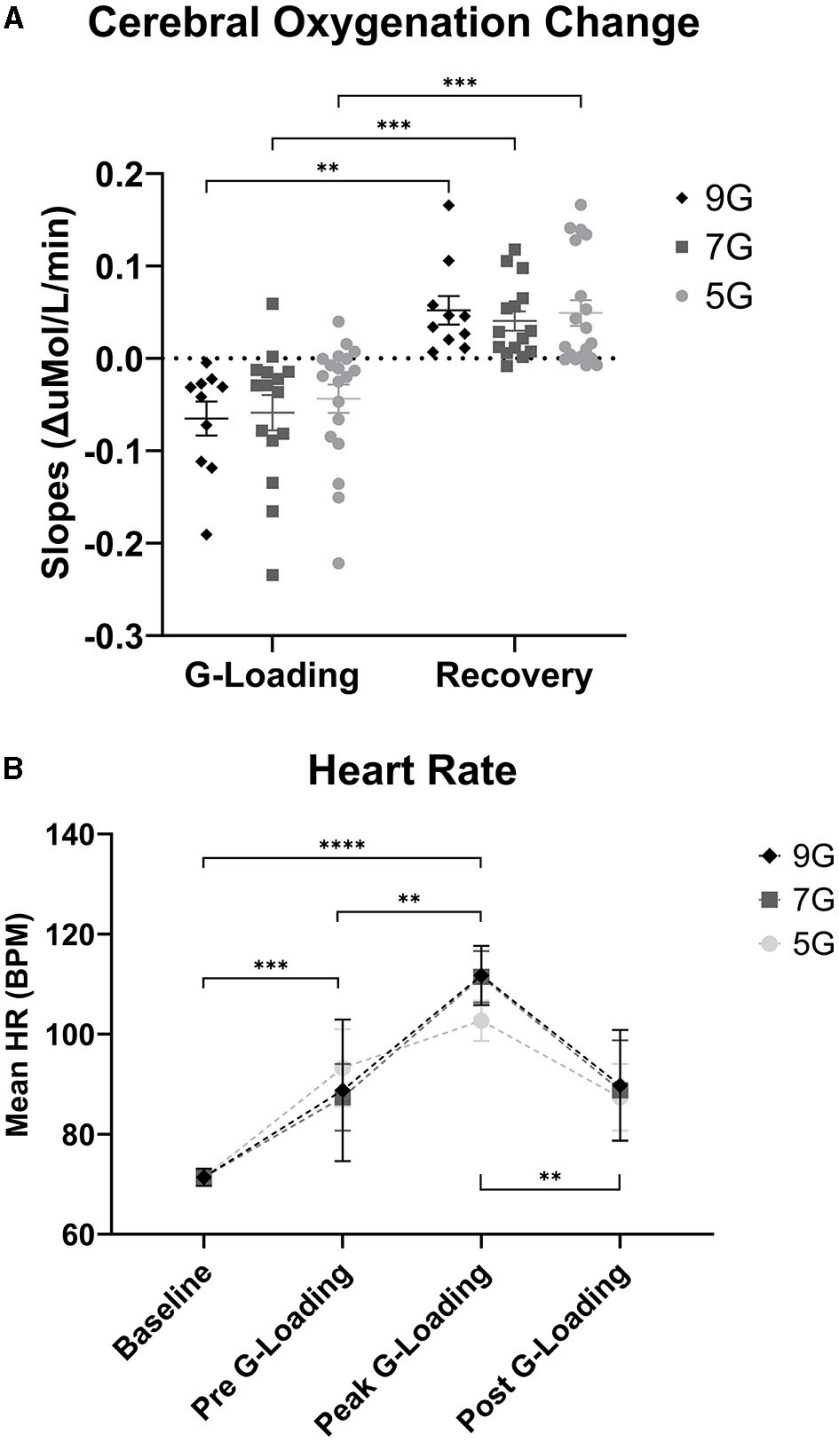
**(A)** Cerebral Oxygenation Trend Slopes during G-loading and Recovery post-G-loading. **(B)** Heart Rate (Polar) data collected during G-exposures. Errors bars represent the standard error of the mean. Asterisks indicate a significantly difference at the group level ([*] = *p* < 0.05, [**] = *p* < 0.01, [***] = *p* < 0.001, [****] = *p* < 0.0001).

Further examination of the data revealed differences in cerebral deoxygenation kinetics between G-loading and Recovery but not between G-exposure levels. As expected, total oxygenation slopes during G-loading were largely negative and were largely positive during recovery post-G-loading (Figure 8A). There was a significant main effect of G condition (loading or recovery) [F_(1, 18)_ = 39.28, *p* < 0.0001], while G level (5G, 7G, or 9G) and their interaction were not significant in the mixed-model analysis. It is possible that the additional G-loading above 5G does not fundamentally affect the cerebral deoxygenation and reoxygenation response in a way that can be meaningfully captured by NIRS. However, the small sample size and heterogeneous experience levels in this human cohort limit interpretation of this outcome. At the individual level, characterizing the rate of deoxygenation during G-exposure in real time may still be particularly relevant in predicting the risk of G-LOC (Eiken et al., 2017). Finally, it is important to note that the current device did not measure “absolute” cerebral oxygenation using the spatially-resolved spectroscopy method (i.e., capable of reporting a regional oxygen saturation or tissue oxygen index parameter) (Suzuki et al., 1999) but estimated Oxygenation as the difference between Oxy-Hb and Deoxy-Hb. While the Aerie's fNIRS-derived metrics do not allow absolute measurements of cerebral oxygenation, trend measurements are sufficient for continuous monitoring of deoxygenation kinetics during G-exposure.

### 3.3 Heart rate measurement outcomes (Polar chest band)

There was a significant main effect of G-loading on HR [F_(3, 115)_ = 25.43, *p* < 0.0001] while G-level or the interaction between G-level and G-loading were not significant. Mean HR at baseline was 71.4 +/ −7.3 BPM, mean HR during 5G exposures was 102.7 +/ −12.1 BPM, mean HR during 7G Exposures was 111.5 +/ −16.3 BPM, and mean HR during 9G exposures was 111.7 +/ −13.2 BPM (Figure 8B). At the group level, there were not differences in HR between 5G, 7G, and 9G. As expected, HR increased during G-loading as subjects underwent severe physiological stress, consistent with a previous report (Ueda et al., 2015). Subjects were also instructed to perform the AGSM maneuver during G-loading which by itself elicits cardiovascular activation (Perry and Lucas, 2021).

## 4 Conclusion

The NIRSense Aerie fNIRS system was specifically designed to enable continuous monitoring of superficial and cerebral oxygenation kinetics of HPA pilots during acute and repeated G-exposure in flight. Its ultra-thin, flexible optical frontend design allows for compatibility with HPA helmets. Despite some challenges, the Aerie demonstrated promising results to detect and alert users of reduction in cerebral and superficial tissue oxygen during G-loading. In a human centrifuge protocol, NIRSense Aerie was able to capture and characterize cerebral deoxygenation responses under high +G acceleration. Pulse oximetry observations showed that acute, high G-loading instantaneously compromised SpO_2_ and PR measurements (due to low/no pulsatile blood measured) suggesting that reliable, continuous pulse oximetry may not be feasible during G-loading periods in these specific recording conditions. On the other hand, uninterrupted cerebrovascular monitoring using fNIRS-based oximetry remains possible during acute G-loading. Subjective user feedback on system fit and feel was generally positive. Future steps for this technology will focus on miniaturization for better interoperability with HPA helmets for maximal aircrew comfort and wearability as well as electronics improvements for ambient light noise rejection and various optical signal optimizations. Future research directions involve the elaboration of a real-time cerebral oxygenation feedback protocol during centrifuge training to improve AGSM learning and retention.

## Data availability statement

The raw data supporting the conclusions of this article will be made available by the authors, without undue reservation.

## Ethics statement

The studies involving humans were approved by IRB #FWR20220101H AFRL IRB 711 HPW/IR 2245 Monahan Way, Bldg 29, Rm 201 Wright-Patterson AFB, OH 45433. The studies were conducted in accordance with the local legislation and institutional requirements. The participants provided their written informed consent to participate in this study.

## Author contributions

TR: Conceptualization, Data curation, Formal analysis, Investigation, Methodology, Project administration, Resources, Software, Supervision, Validation, Visualization, Writing—original draft, Writing—review & editing. RB: Conceptualization, Funding acquisition, Methodology, Project administration, Resources, Supervision, Writing—original draft, Writing—review & editing. JS: Methodology, Writing—original draft, Writing—review & editing. JA: Methodology, Software, Validation, Writing—original draft, Writing—review & editing. BG: Methodology, Software, Validation, Writing—original draft, Writing—review & editing. NM: Methodology, Validation, Writing—original draft, Writing—review & editing. TS: Methodology, Software, Writing—original draft, Writing—review & editing. SC: Methodology, Writing—original draft, Writing—review & editing. BM: Conceptualization, Data curation, Investigation, Methodology, Project administration, Supervision, Visualization, Writing—original draft, Writing—review & editing. JB: Investigation, Methodology, Project administration, Resources, Writing—original draft, Writing—review & editing. PS: Funding acquisition, Project administration, Resources, Supervision, Writing—original draft, Writing—review & editing.
